# A Reviewed article: Silva LAN, Delpino FM, Figueiredo LM, Thumé E, Tomasi E, Facchini LA, Nunes BP. Adult use of health services: a cross-sectional population-based study, Pelotas, Brazil, 2012 and 2021. Epidemiol Serv Saude.2026:35;e20250460.

**DOI:** 10.1590/S2237-96222026v35e20250460.b

**Published:** 2026-04-20

**Authors:** Isiyara Taverna Pimenta

**Affiliations:** 1 Secretaria Municipal de Saúde do Rio de Janeiro, Rio de Janeiro, RJ, Brazil Secretaria Municipal de Saúde do Rio de Janeiro Rio de Janeiro RJ Brazil

## Peer review A

## Questionnaire

Does the manuscript contain new and significant information to justify publication? Yes

Does the Abstract (Summary) clearly and accurately describe the content of the article? No

Is the problem significant and concisely stated? No

Are the methods described comprehensively? No

Are the interpretations and conclusions justified by the results? No

Is adequate reference made to other work in the field? Yes

## Manuscript Structure:

Length of article is: Too little

Number of tables is: Adequate

Number of figures is: Adequate

Please state any conflict(s) of interest that you have in relation to the review of this paper: None

Interest: Average

Quality: Average

Originality: Average

Overall: Average

Recommendation: Average

## Comments

Obrigada pela oportunidade de revisar este manuscrito. Encaminho os meus comentários e sugestões para auxiliar na melhoria da qualidade do artigo.

1) Resumo

- Métodos: Como foram avaliadas as desigualdades socioeconômicas? E o acesso aos serviços de saúde? Os autores poderiam detalhar melhor os métodos.

- Conclusão: Deter-se ao que se pode afirmar a partir dos resultados obtidos no estudo.

2) Introdução

- Os autores citam que estudos realizados em países de alta renda e no Brasil apontaram para a redução do acesso aos serviços de saúde, no entanto, não especificam os períodos avaliados nesses estudos e nem as características dos serviços de saúde dos locais onde foram realizados (públicos, privados etc.). Importante acrescentar essa informação.

- Por que é importante avaliar a utilização dos serviços de saúde considerando os anos 2012 e 2021? Senti falta de uma justificativa robusta da relevância do estudo.

- Importante trazer também uma problematização a respeito da pandemia de COVID-19, uma vez que 2021 foi um ano pandêmico.

- Os autores também precisam contextualizar as desigualdades socioeconômicas, por que são importantes no contexto do acesso aos serviços de saúde?

- Como é a estrutura do setor de assistência à saúde de Pelotas? Houve mudanças considerando os anos 2012 e 2021? Detalhar.

3) Métodos

- As pesquisas que foram a base de dados do estudo são representativas da população de Pelotas? Citar maiores informações sobre o processo de amostragem.

- As amostras dos dois estudos possuem características distintas, um estudo incluiu todos os residentes dos domicílios selecionados com 10 anos ou mais, outro estudo incluiu um residente do domicílio selecionado e com 18 anos ou mais. Considerando que o objetivo é comparar o acesso aos serviços de saúde nas duas amostras, pode-se considerar que elas são de fato comparáveis? Há potencial para viés?

- A pergunta que avaliou o acesso aos serviços de saúde se referiu a que dia do mês passado? O mesmo dia da realização da entrevista? Não ficou claro.

- Por que os autores juntaram as raças parda, amarela e indígena na mesma categoria? Justificar.

- Descrever com detalhes como foi feita a classificação econômica e como foi avaliada a percepção de saúde.

- Nos métodos estatísticos os autores citam que avaliaram a classe social, mas isso não foi citado anteriormente, na descrição das variáveis.

- Não ficou claro como feito o cálculo do Índice Absoluto de Desigualdades e nem a aplicabilidade da regressão logística para esse fim. Os autores precisam explicar melhor e acrescentar no tópico “Variáveis de mensuração”.

4) Resultados

- No trecho “Houve uma associação inversa entre escolaridade e utilização dos serviços de saúde, estatisticamente significativa apenas em 2012”, os autores fizeram análise de associação? Não foi citado no método, como chegaram a esse resultado?

- “As análises de sensibilidade realizadas para 2012 mostraram resultados semelhantes, considerando tanto todos os moradores do domicílio quanto apenas um morador selecionado, com aumento da prevalência de utilização de 29,3% para 30,7%. No entanto, não foram observadas diferenças significativas para idade e escolaridade. Em relação a 2021, as análises realizadas sem a ponderação do desenho amostral (o que resultou em maior representação de mulheres e idosos na amostra) indicaram um aumento da prevalência de utilização de 34,4% para 36,5%, mantendo um padrão similar de desigualdades.” Quais são os intervalos de confiança das estimativas? Por que as análises foram realizadas sem ponderação do desenho amostral? Isso confere potencial para viés de informação.

5) Discussão

- Quais são as limitações do estudo? Acrescentar

